# Removal of Pb(II), Cd(II) and Ni(II) Ions from Groundwater by Nonthermal Plasma

**DOI:** 10.3390/ma15155426

**Published:** 2022-08-06

**Authors:** Beata Jabłońska, Tomasz Dróżdż, Paweł Jabłoński, Paweł Kiełbasa

**Affiliations:** 1 Faculty of Infrastructure and Environment, Czestochowa University of Technology, Brzeźnicka St. 60a, 42-200 Częstochowa, Poland; 2 Faculty of Production and Power Engineering, University of Agriculture in Krakow, Al. Mickiewicza 21, 31-120 Krakow, Poland; 3 Faculty of Electrical Engineering, Czestochowa University of Technology, Al. Armii Krajowej 17, 42-200 Częstochowa, Poland

**Keywords:** low-temperature plasma, nonthermal plasma, cold plasma, heavy metal removal, dielectric barrier discharge, water treatment

## Abstract

The removal of Pb(II), Cd(II) and Ni(II) ions from aqueous solutions by means of nonthermal plasma with a dielectric barrier discharge is investigated. Aqueous solutions with metal ion concentrations from 10 to 100 mg/dm^3^ in spring water were used. In the first stage, the optimization of the solution flow rate, generator modulation frequency and duty cycle was made in terms of the removal efficiency of the considered metals. The removal was then investigated as a function of the number of passes of the solution through the cold plasma reactor. The effect of the initial concentration of ions in the solution was studied. Techniques such as composite central design, least squares method and Fourier transform infrared spectroscopy were used. The physical and chemical parameters of the solutions, such as electrical conductivity, pH, temperature, concentration of metal ions and the content of other substances (e.g., total organic carbon), were measured, and the presence of microorganisms was also examined. It was found that each pass of the solution through the cold plasma reactor causes a decrease in the concentration of Cd(II) and Ni(II); the concentration of Pb(II) drops rapidly after one pass, but further passes do not improve its removal. The removal percentage was 88% for Cd(II) after six passes and 72% for Pb(II) after one pass, whereas 19% for Ni(II). The purification mechanism corresponds to the precipitation of metal ions due to the increasing pH of the solution after exposure to cold plasma.

## 1. Introduction

Contamination of surface water and groundwater with trace elements is one of a worldwide challenges due to their high toxicity, persistence and high level of bioaccumulation in the environment [1,2]. The quality of surface waters has been adversely affected by continuously progressing industrialization, urbanization and the mismanagement of water resources [3]. Among the substances polluting natural waters, heavy metal ions belong to most frequently released, together with sewage, waste and dust. The main sources of pollution of the aquatic environment with these metals are emissions from industrial plants, metal smelters, atmospheric precipitation, leachate from landfills and industrial and municipal sewage from urbanized areas. Source of lead can also include gasoline, household paints, batteries and zinc-lead ores [4,5]. Once introduced into the environment, they remain there and interact for a long period of time due to the negligible degree of degradation [6]. They are non-degradable pollutants that are not subject to significant chemical and microbiological transformations.

The harmfulness of heavy metals polluting the environment is largely due to their biochemical and biological properties. Heavy metal ions, such as lead, nickel and cadmium, play no role in cell metabolism, but are easily absorbed and stored in various parts of plants and living organisms [4,7]. In general, they easily cross biological membranes and form bonds with proteins, nucleic acids and lipids, causing various types of cell damage and disturbance of their metabolic functions [8]. Heavy metal toxicity also disrupts the supply of water and nutrients from the soil to the upper parts of plants and degrades the activity of leaf pigments, such as the chlorophyll content [9]. For example, in plants, lead and cadmium in excessive concentrations (100–400 mg/kg depending on the species) cause changes in physiological processes that lead to growth inhibition and reduction in nutrient uptake, which in turn causes the development of chlorosis and necrosis. This phenomenon is associated with iron deficiency and inhibition of photosynthesis [10,11]. The toxic effect of Ni(II), Cd(II) and Pb(II) on the body is manifested by allergic reactions, disturbances in protein metabolism in the plasma and changes in chromosomes and bone marrow [8]. 

Due to the inevitable emissions of pollutants into the environment, it is necessary to remove them in order to meet stringent regulations on the quality of water discharged into aquatic ecosystems. The sustainable management of water resources and the constantly deepening water deficit in the world forces the search for new technologies of wastewater treatment and the use of closed water circuits [12,13]. Currently, various methods are used to remove heavy metals from water, such as precipitation [14], adsorption [11,15,16,17], ion exchange [18], reverse osmosis [19], membrane filtration [20], electrolysis [21] and others [3]. However, many of these methods are associated with recontamination, low efficiency and high costs. Among water and wastewater methods, advanced oxidation processes (AOPs) are becoming more and more important in the degradation of hazardous and difficult-to-decompose pollutants [22,23,24,25]. A common feature of AOPs is the generation of a wide spectrum of reactive molecular forms, such as free electrons, H^•^, O, ^•^OH, OH^−^, H^+^, HO_2_^−^, HO_2_^•^, H_3_O^+^, O^•−^, O_2_^•−^, O_3_^−^, H_2_O_2_, O_3_ and H_2_O* [26]. The species easily react with various chemical substances, which allows for the decomposition of toxic compounds, modification of polymer structures, destruction of bacteria and fungi and influencing their biological structures and DNA [27,28]. One of the methods used in AOPs is the generation of plasma. In addition to reactive molecular forms, plasma technologies are able to produce shock wave, UV light quanta and electrohydraulic cavitation [22,27,29,30]. Electrical discharges in plasma above or in a liquid also initiate redox processes [31]. Therefore, plasma-based processes are generally considered to be combined with other AOPs, including ozonation, UV photolysis and pyrolysis [32]. They have the advantages of not requiring temperature and pressure and being energy saving and ecological, and therefore environmentally friendly [22]. Among plasma technologies, nonthermal plasma (NTP) with dielectric barrier discharges (DBDs) is often used for water treatment [33]. This technology uses thin-film reactors, usually consisting of two coaxial quartz tubes and a disc and mesh electrodes made of stainless steel [34]. In systems of this type, a large number of oxidizing species with a short lifetime is formed, in contrast to the chemical reagents used in other APO methods [25].

In the last few years, there has been increased interest in using plasma technology to reduce water pollution [35]. Currently, researches on using this technology mostly concern removing organic compounds from water and wastewater, including dyes [36,37], pharmaceuticals [38,39], phenol [40], methylene blue [41], phosphorous compounds [42] and microbes [43]. Plasma technologies can also be used to oxidize inorganic pollutants, but the number of articles on this application is small [23,44]. In addition, plasma techniques can also be used in conjunction with other water and wastewater treatment processes [29]. However, they have not been tested for the removal of metal ions in treated water. Some inorganic pollutants under the action of oxidizing agents change, which facilitate their precipitation in the form of sediments or electrocoagulation [45]. Plasma reactivity is strictly dependent on the type of plasma generators used (impulsive, DC, DBD, fluorescent and others) and their parameters as well as the physical and chemical properties of the treated water or sewage (conductivity, pH and the content of organic and inorganic compounds in them). Therefore, plasma technologies should be further explored to determine their properties and optimize treatment processes [22].

The aim of the study is to investigate whether Pb(II), Cd(II) and Ni(II) in concentrations comparable with those in industrial wastewater can be removed from aqueous solutions via nonthermal plasma with a dielectric barrier discharge. An attempt was also made to determine the effect of NTP on the quality of the tested water as well as the effect of initial concentration of heavy metals and the pH of the solution on removing the pollutants from the water. The optimization of cold plasma generator settings was also carried out in terms of the effectiveness of metal ion removal.

## 2. Materials and Methods

### 2.1. Lab Stand

**Figure 1:**
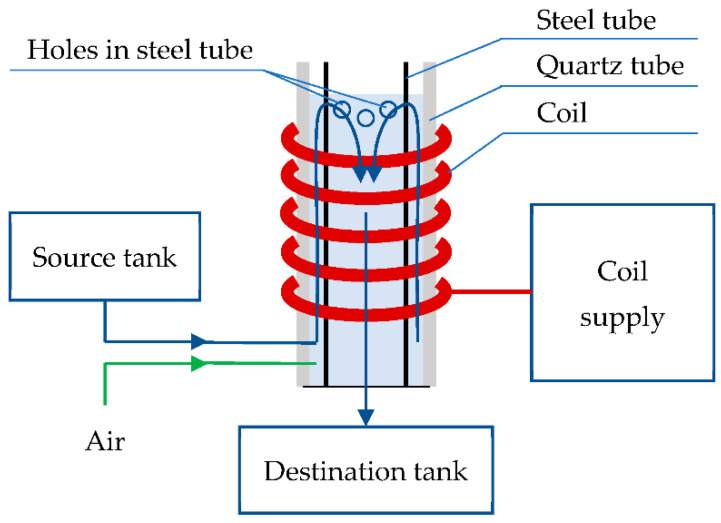
Schematic diagram for the nonthermal plasma reactor with dielectric barrier discharges.

**Figure 2:**
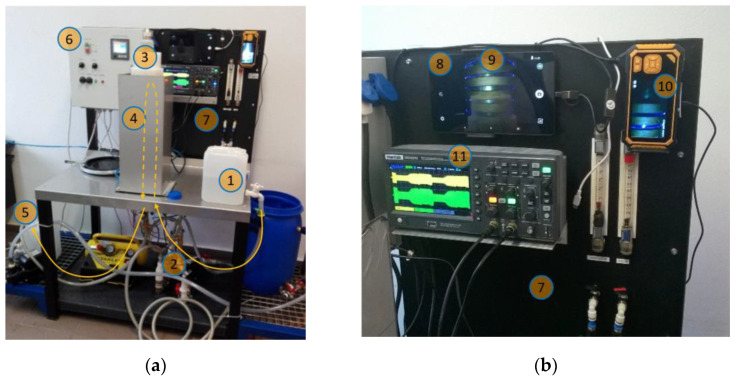
Experimental lab stand with nonthermal plasma reactor (yellow arrows indicate the direction of water flow). (**a**) Metering and visualization panel; (**b**) 1—source tank, 2—pump, 3—quartz tube enclosed in a case, 4—metallic shield against electric field, 5—destination tank, 6—supply panel, 7—control and visualization panel, 8—main monitor, 9—coil visible on the main monitor, 10—auxiliary monitor (visible coil turns and blue light of discharges), 11—scope with visible voltage and current waveforms in the coil.

The research was carried out using an experimental device with a NTP reactor with DBDs. The schematic diagram is shown in Figure 1, and the lab stand is presented in Figure 2. On a quartz tube, a coil is wound; one of its terminals is connected to AC voltage of 7.5 kV and a frequency of 75 kHz. This voltage is excited with a selected modulation frequency of 100–400 Hz. Inside the quartz tube, a conductive tube is placed. The water to be treated as well as air, which is mixed with the water, is supplied through the lower part of the quartz tube. The aerated water flows between the two pipes and is then directed into a destination tank. From an electrical point of view, the arrangement of the coil, quartz tube, water and metal tube is an LC circuit. When operating in a resonant state, it can generate a high electric field strength in the working area between the coil turns and the metal tube. This field causes electric discharges inside air bubbles and the formation of UV radiation, whose photons have enough energy to ionize or excite water molecules, oxygen and other compounds present in the polluted water. The device has the option of setting the flow rate through the reactor, modulation frequency and duty ratio.

### 2.2. Physical, Chemical and Biological Analyses

As reported in [46], electrical discharges in demineralized water are weak; therefore, water taken from a spring located in the southern part of Poland was used for the research. Spring water contains a certain number of cations, e.g., sodium, calcium or potassium, and anions, e.g., bicarbonate and sulphate. The presence of ions in the tested water facilitates the formation of ^•^OH radicals and OH^−^ ions during the dielectric barrier discharge in the cold plasma [23,26].

The tests were carried out on the day of water intake. In the tested water, pH, turbidity, electrolytic conductivity, total hardness, oxidizability, total organic carbon (TOC), ammonium ions, nitrates, nitrites, chlorides, sodium, calcium, sodium and trace elements, such as total Fe, Mn, Mg, Cd, Pb, Cu, Ni and total Cr, were found. The analyses of the individual physical and chemical properties of the tested water were determined using the reference methods indicated in [47]. Each determination was performed five times and each measurement series was carried out in the same way. The pH measurements were carried out with an ELMETRON pH/mv CP-401 pH meter with an accuracy of ±0.002. Turbidity (NTU) was measured with a Hach 2100N turbidimeter. Total organic carbon was determined on an N/C 3100 multi analyzer (Analytik Jena, Jena, Germany) equipped with a TC + TIC module. The specific electrolytic conductivity was determined using a G1409-L01 conductometer by Greisinger according to the standard [48]; total water hardness according to [49]; permanganate index according to [50]; ammonium ions according to [51]; nitrates according to [52]; nitrites according to [53]; calcium, magnesium and chlorides according to [54]; and sodium according to [55]. Nitrogen and sodium compounds were determined with a UV-VIS DR 5000 spectrophotometer by Hach-Lange. The content of heavy metals (total Fe, Mn, Mg, Cd, Pb, Cu, Ni and total Cr) was determined using an inductively coupled plasma atomic emission spectrometer (ICP-AES) with Spectro Arcos system (SPECTRO Analytical Instruments, Kleve, Germany).

The microbiological tests included the determination of the total number of microorganisms, coliform bacteria, *Escherichia coli* and *Enterococcus*. The total number of microorganisms (also referred to as the number of heterotrophic bacteria, total number of bacteria or number of colonies) was determined according to the standard of [56] by the deep plating method on agar with a yeast extract at a temperature of 22 ± 2 °C during 72 h of incubation. *Enterococcus* bacteria were determined according to [57], while bacteria of the coli group and *Escherichia coli* according to [58]. The basis of the method is cultivation in a liquid medium, and the value of the most probable number (MPN) of the organisms sought is read from the relevant MPN tables.

### 2.3. Removing Metal Ions

The experiments were carried out on synthetic solutions prepared on the basis of chemical compounds of metals (analytical grade) containing the single metal ions cadmium, nickel and lead in initial concentrations comparable to those in industrial wastewater. The tested water was contaminated with heavy metals in concentrations from 10 to 100 mg/dm^3^. The metals of high mobility in the environment, i.e., Cd(II), Ni(II) and Pb(II), were selected for the study, because they often occur locally in surface and underground waters [59]. A total of 5 L of water contaminated with the specified metal were passed through the plasma reactor each time. The research included: the optimization of the device settings, examination of the effect of the number of cleaning cycles and examination of the effect of the initial concentration of metal ions on the removal efficiency.

In the first stage, the focus was set on selecting the generator settings. Their influence can be roughly estimated from the following mathematical model. Let the flow rate be p [dm^3^/min], the modulation frequency $f_{m}$ [Hz] and the duty cycle d. Let the volume of the working space (between the quartz tube and the metal tube) be V [dm^3^]. The time needed for the displacement of this volume of water through the working space is V/p [min]. However, the duration of the discharge is actually shorter due to the duty cycle d, and equals $T_{\mathrm{eff}}=dV/p$. Therefore, the effective time during which the water is affected to discharges per unit volume of water is $t_{\mathrm{eff}}=T_{\mathrm{eff}}/V=d/p$ [min/dm^3^]. The greater the effective time of interaction per unit volume, the greater the expected effect. Therefore, the duty cycle should be as high as possible and the flow rate as low as possible. The above analysis shows that the modulation frequency is not an important factor; however, this analysis does not take into account other factors, such as air flow and the frequency of discharges. Therefore, the measurement results may differ from the predictions. As a result, it was decided to determine the optimal flow rate, modulation frequency and duty cycle. The influence of these settings on the removal of Pb(II), Cd(II) and Ni(II) ions with an initial concentration of 10 mg/dm^3^ was investigated. The central composite design method in the inscribed version (CCI) was used for three variables with the method parameter α = 23/4 ≈ 1.68. The flow rate was tested in the range of 0.5–2.5 dm^3^/min, modulation frequency was in the range of 100–400 Hz and duty cycle was 10–90%. These variables were normalized according to the CCI rules and denoted $x_{1}$, $x_{2}$ and $x_{3}$, respectively, as in Table 1.

For each of the three tested metals Pb(II), Cd(II) and Ni(II), aqueous solutions with a concentration of 10 mg/dm^3^ were prepared. Spring water with an initial pH of 7.57 and a temperature of 22 °C was used. After mixing and allowing it to equilibrate, the pH and the metal concentration were measured. The solution was then subjected to the NTP reactor (one pass) for various settings of flow rate, modulation frequency and duty cycle according to the 20 points CCI scheme for three variables. The pH and the concentration of metal ions were measured. Then, the pH change and the percentage of ion removal were calculated as follows:

(1) $$\Delta \mathrm{pH}=\mathrm{pH}-{\mathrm{pH}}_{\mathrm{initial}},$$

(2) $$\mathrm{Removal}\;\left[\%\right]=\frac{C_{\mathrm{initial}}-C_{\mathrm{final}}}{C_{\mathrm{initial}}}\times 100\%,$$

where $C_{\mathrm{initial}}$ = 10 mg/dm^3^ and $C_{\mathrm{final}}$ is the final concentration of metal ions. The determined values of ΔpH and Removal were then used to find the best fit to a function of the following form:

(3) $$a_{0}+a_{1}x_{1}+a_{2}x_{2}+a_{3}x_{3}+a_{4}x_{1}^{2}+a_{5}x_{2}^{2}+a_{6}x_{3}^{2}+a_{7}x_{1}x_{2}+a_{8}x_{1}x_{3}+a_{9}x_{2}x_{3}$$

The unknown coefficients $a_{0},a_{1},\ldots ,a_{9}$ were found with the least squares method, taking the adjusted *R*^2^ as a measure of the fitting, which penalizes the number of degrees of freedom. The adjustment was performed for all possible combinations of base functions, and the best match was assumed that one that had the highest value of adjusted *R*^2^. This allowed to eliminate the terms that had only a very insignificant influence on the values of the explained variable. Further tests were carried out for a solution flow of 0.5 dm^3^/min, a modulation frequency of 300 Hz and 90% duty cycle—these parameters were selected as a result of the optimization of the settings from the above-described test stage.

In the next stage, solutions of Pb(II), Cd(II) and Ni(II) ions with an initial concentration of 10 mg/dm^3^ were prepared, and studies on the removal of Pb(II), Cd(II) and Ni(II) ions were carried out depending on the number of passes of contaminated water through the plasma reactor. The change in pH and the percentage removal of Pb(II), Cd(II) and Ni(II) ions were investigated. In addition, the temperature of the solution was measured after each pass as well as the energy intake was traced. In addition, the FTIR analysis of the samples obtained after 6 passes was performed.

Apart from the above, the effect of the concentration of Pb(II), Cd(II) and Ni(II) ions was investigated. The concentrations varied in the range of 10–100 mg/dm^3^, and the solutions were passed once through the reactor. The change in pH and the percentage removal were determined, as well as temperature and energy intake being measured.

The energetical efficacy of metal ions removal was calculated as follows:

(4) $$E=\frac{\left(C_{\mathrm{initial}}-C_{\mathrm{final}}\right)V}{W},$$

where V is the solution volume and W is the energy intake.

## 3. Results and Discussion

### 3.1. Characteristics of the Used Water

The overall characteristics of the water used in the tests along with the values of the permissible values for pollutants discharged into water and soil as well as industrial wastewater are presented in Table 2 and Table 3. The conducted research shows that the parameters of the tested water do not exceed the permissible values for surface waters.

From a bacteriological point of view, no pathogenic microorganism from the coliform bacteria, including *Escherichia coli* and *Enterococcus*, were found in the tested water. On the other hand, the total number of microorganisms was over 300 CFU and exceeded twice the limit values for tap water [60]. Most of the bacteria that are hazardous to human health belong to the mesophilic group, for which the human body is an excellent incubator due to the optimal temperature for development. Therefore, an increase in the total number of bacteria is always a warning signal of a deterioration in water quality and a health hazard [61].

### 3.2. Optimization of the NTP Generator Settings

The results of the research regarding the selection of the optimal settings of the cold plasma generator, solution flow rate through the reactor, modulation frequency and duty cycle are presented in Table 4 and Table 5.

Table 5 shows that the variance of the examined indicators (ΔpH and Removal) is not very well explained by the assumed approximation (adjusted *R*^2^ in the range of 0.40–0.76). This is probably due to the high content of the random component and the measurement uncertainty. However, it can be seen that after one pass of the water through the reactor, the pH increased, with the average increase being the greatest for Ni(II) and the smallest for Cd(II). Nevertheless, it is difficult to say which of the explanatory variables has the dominant contribution. It is noticeable that an increase in $x_{1}$ (flow rate) contributes negatively to the increase in pH, which is in line with the above-mentioned prediction, as the higher the flow rate, the shorter the time of plasma impact on the water. The effect of the remaining variables (modulation frequency and duty cycle) is not unequivocal, although it would be expected that a high duty cycle should result in a stronger impact on water and a greater increase in pH. Moreover, the influence of the tested generator settings on the removal of metal ions is not obvious, although a decrease in concentration is observed in each case, i.e., a positive effect on removal. Regarding the removal of Pb(II), it should be noted that the most of the lead had precipitated out of the solution before exposure to cold plasma, and the subsequent changes in Pb(II) concentration were insignificant. The greatest changes were observed in the case of Cd(II) and the smallest in the case of Ni(II), but in no case was there a clear effect of the settings on the removal of metal ions.

**Figure 3:**
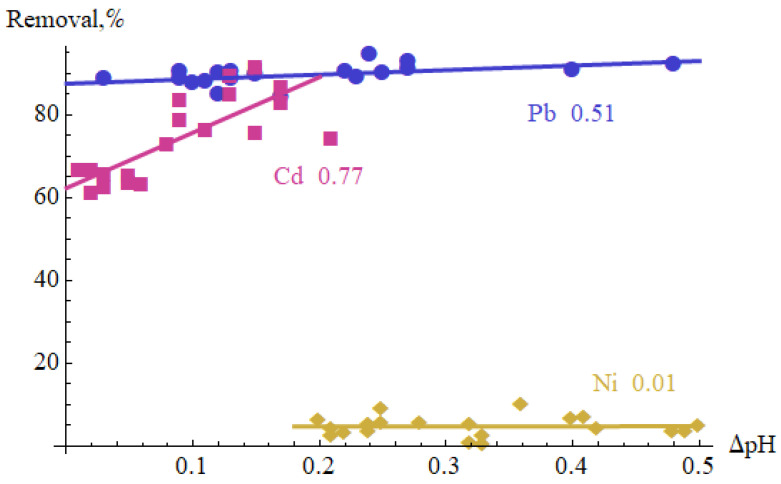
Generator settings optimization results in (ΔpH, Removal) coordinates.

Table 6 shows the Pearson correlation coefficients between flow rate, modulation frequency, duty cycle, ΔpH and Removal. Their absolute values are mostly lower than 0.5 and do not indicate a strong correlation between the generator settings and ΔpH and Removal. There was no significant correlation coefficient between the flow rate and other variables. The modulation frequency has a positive effect on the increase in pH in the case of Pb(II) solution (correlation coefficient 0.56), and the duty cycle on Ni(II) removal (0.59). Figure 3 shows the measurement results in (ΔpH, Removal) coordinates. The highest correlation coefficient between ΔpH and Removal was observed for Cd(II) (0.77), quite a clear correlation occurred for Pb(II) (0.51), while no correlation was found between these variables for Ni(II). This may be due to the fact that Ni(II) precipitates very poorly in the considered pH range.

Based on the above results, it can be concluded that a minimum flow rate is desirable. The research did not show a clear effect of the modulation frequency and duty cycle. This may indicate that long living reactive species are crucial in the treatment mechanism. The theoretical considerations indicate that the modulation frequency itself is not of great importance, but the duty cycle is important, as it directly affects the reactor’s impact time on the water. Therefore, in further tests, the flow rate was set to 0.5 dm^3^/min (lowest possible for the device), the modulation frequency was arbitrarily set to 300 Hz and the duty cycle to 90% (maximum possible for the device).

### 3.3. Physical, Chemical and Microbiological Parameters of the Tested Water

Physical, chemical and microbiological parameters of water samples before and after treatment with cold plasma with DBD discharges are presented in Table 7. It can be seen that the cold plasma increased the electrolytic conductivity by about 2–2.5%. Similar results were obtained in [62]. The increase in conductivity indicates the presence of new ionic forms in the solution resulting from the electric discharge and ionic forms from the destruction of contaminating compounds contained in the water (e.g., H_3_O^+^, NO_2_^−^ and NO_3_^−^). The electrolytic conductivity of liquids is one of the important parameters affecting the intensity of UV radiation (it increases in solutions with higher conductivity) as well as the generation of a shock wave or thermal effects [29]. As reported by [63], an exceedingly high water conductivity is not desirable due to the lower production of ^•^OH radicals and H_2_O_2_ in cold plasma.

The turbidity increased significantly after the first pass, which is due to the turbulent nature of the flow through the cold plasma reactor. After the next passes, the turbidity decreased due to the precipitation of impurities under the influence of various types of reducing forms. There was a noticeable increase in TOC, while the content of inorganic compounds slightly decreased. It is likely that the carbon present in the water, e.g., carbon dioxide dissolved in water, has been partially converted into simple organic substances.

The action of plasma, due to the UV light photons, ozone, radicals and atomic oxygen it contains, also leads to the degradation of microorganisms [62,64]. The synergistic action of these factors effectively reduced the amount of the total number of microorganisms in the tested water after just one pass through the plasma reactor (87%). Further passes through the reactor, i.e., three and six times, destroyed around 95% and 98% of bacteria, respectively, which proves a very good inactivation of microorganisms by the NTP reactor.

### 3.4. Studies on the Removal of Pb(II), Cd(II) and Ni(II)

**Figure 4:**
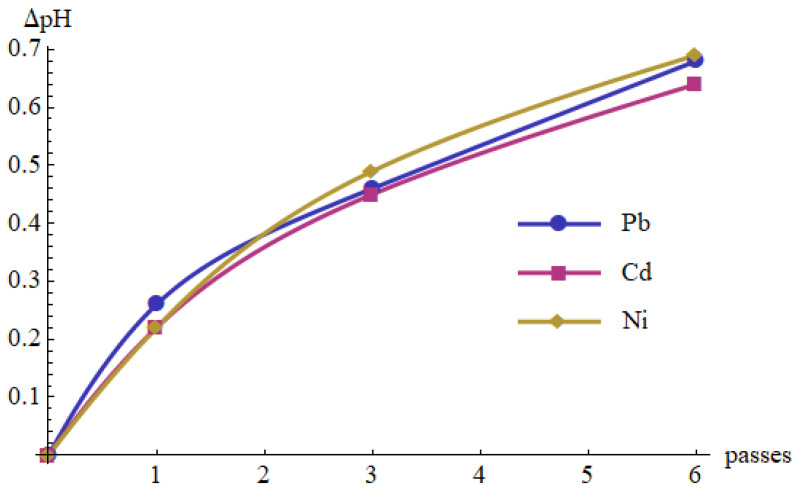
Changes in the pH of the Pb(II), Cd(II) and Ni(II) aqueous solutions vs. number of passes through the cold plasma reactor.

**Figure 5:**
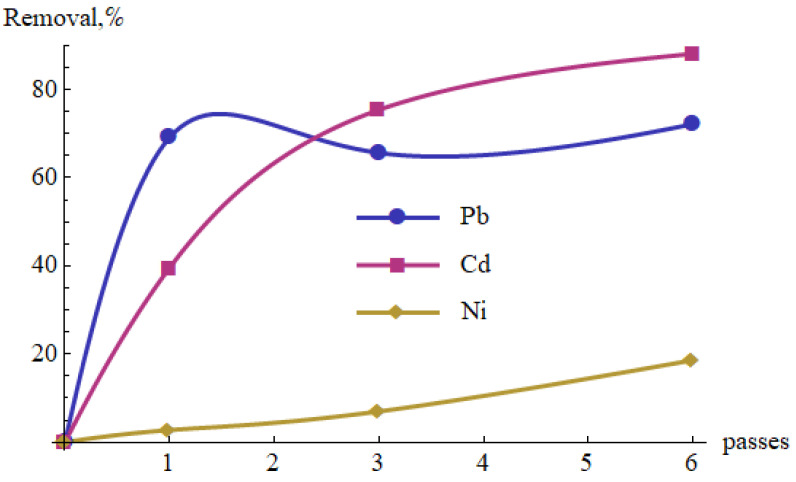
Percentage removal of Pb(II), Cd(II) and Ni(II) from the aqueous solutions vs. number of passes through the cold plasma reactor.

The measurement results are summarized in Table 8 and presented in Figure 4 and Figure 5. As shown in Figure 4, the pH increased after each pass through the cold plasma reactor. After the six passes, the increase was approximately 0.65. Growth weakened in subsequent cycles. Moreover, the percentage of metal removal tended to increase with the number of cycles (Figure 5). It was observed that Pb(II) concentration decreased after the first pass, then increased slightly after the third pass, to decrease again after the sixth pass. These changes, however, were not large and may result from the turbulent nature of the solution flow and the related fluctuations in the local values of Pb(II) concentration. It is also possible that some components of the already precipitated phases re-ionized at a certain pH and entered the dissolved phase.

**Figure 6:**
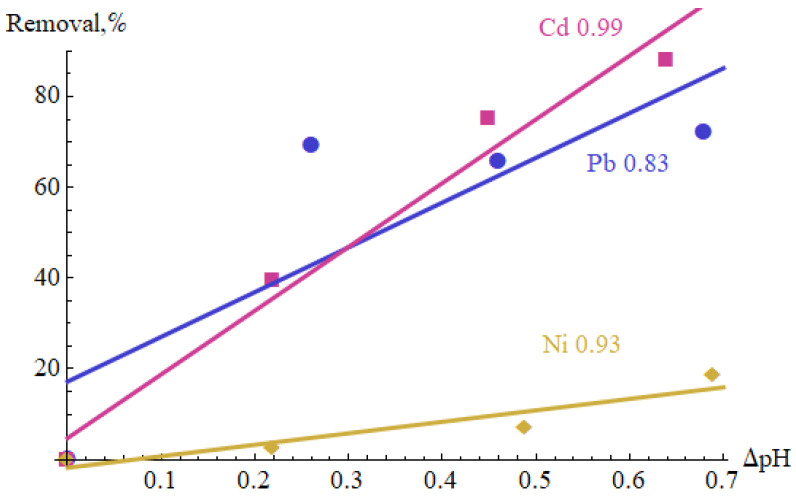
Correlations between pH changes and percentage removal versus number of cycles.

The percentage removal of the metal ions increased along with the number of passes (Figure 5). It reached 72% after one pass for Pb(II) and 88% after six passes for Cd(II), whereas only around 19% for Ni(II). Figure 6 shows the results of the measurements in the coordinate system (ΔpH, Removal). The values of the correlation coefficients between these two variables are also shown. Their values range from 0.83 to 0.99.

Since pH has a great impact in the precipitation of the considered metals, it follows that pH values were too low for the effective precipitation of Ni(II) compounds. The lower removal of Ni(II) than Cd(II) is rather strange, because most references indicate that Ni(OH)_2_ precipitates at pH slightly lower than that for Cd(OH)_2_. For example, the theoretical solubility diagram indicates that Ni(OH)_2_ precipitates at pH around 1 lower than that for Cd(OH)_2_ [65], but in [66] the difference is around 0.5. According to [67], the precipitation of Pb(OH)^+^ occurs in the pH range of 4–11, and Pb(OH)_2_ starts at a pH of around 8, whereas the precipitation of Cd(OH)^+^ occurs in the pH range of 7–12, and Cd(OH)_2_ starts at a pH of around 8. As for Ni(OH)^+^ and Ni(OH)_2_, the starting pH is around 7 and 8, respectively [68]. According to [69], the precipitation of Cd(II), Ni(II) and Pb(II) hydroxides occurs for pH 7–14, 8–14 and 7–8, respectively. Thus, the differences between pH for Ni(II) and Cd(II) are not great, and the presence of other factors, e.g., specific ions and other forms due to cold plasma, could change the pH values as well as influence the formation of other compounds [68]. This requires further research.

In addition, an increase in water temperature is visible in the subsequent passes (Table 8). After the first pass, the temperature rise was 2–2.5 °C. In the subsequent passes, the increase was lower due to the increasing losses related to the transfer of heat from the solution to the device components. After six passes, the temperature increased by about 8–8.5 °C. The increase in water temperature is a visible symptom of energy transfer to water, although from the point of view of water purification, it is not a favorable phenomenon—a higher temperature usually increases the solubility of the compounds, which opposes their precipitation.

**Figure 7:**
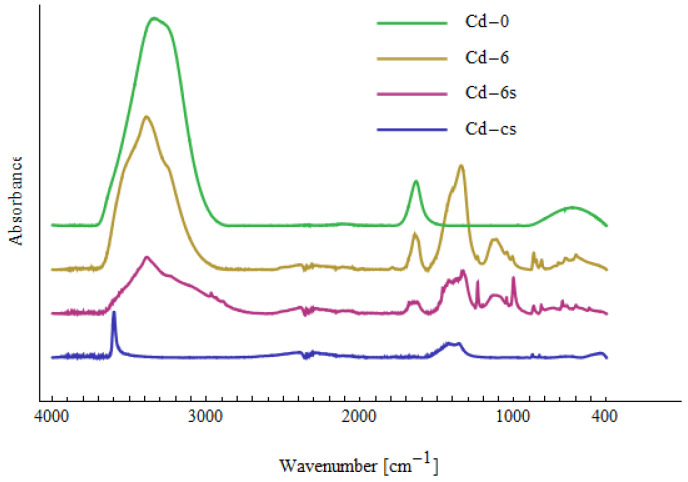
FTIR spectra related to solutions containing Cd(II).

**Figure 8:**
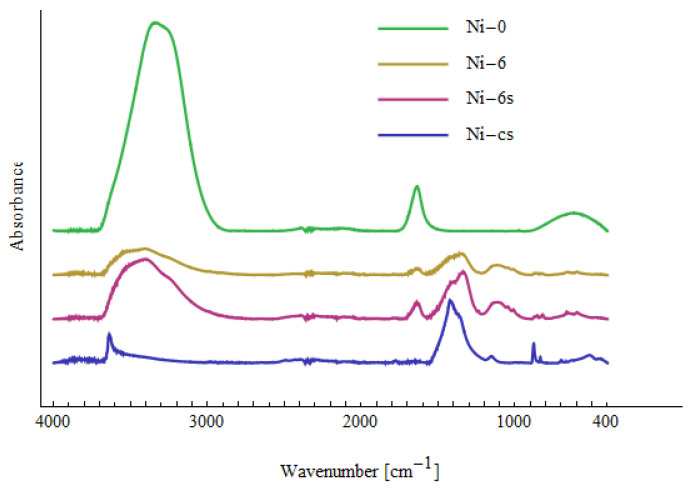
FTIR spectra related to solutions containing Ni(II).

**Figure 9:**
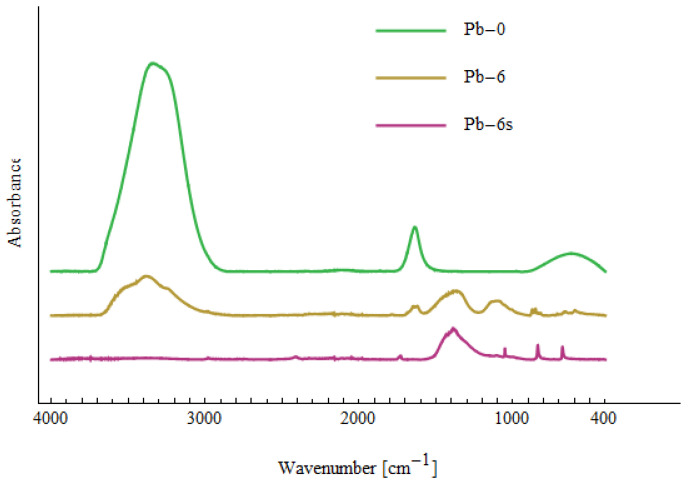
FTIR spectra related to solutions containing Pb(II).

Figure 7, Figure 8 and Figure 9 show the Fourier transform infrared (FTIR) spectra of the tested samples. The samples Cd-0, Ni-0 and Pb-0 relate to the prepared solutions of Cd(II), Ni(II) and Pb(II) with a concentration of 10 mg/dm^3^ before treatment with NTP. All three spectra look very similar. There are three peaks characteristic for water and corresponding to O-H bond stretching (around 3300 cm^−1^), H_2_O bending (1640 cm^−1^) and O-H bending (620 cm^−1^) [70]. The lack of peaks characteristic for bonds with Cd(II), Ni(II) or Pb(II) indicates that no significant amounts of complexes containing the above-mentioned compounds were found in the solution.

The samples Cd-6, Ni-6 and Pb-6 relate to solutions after six passes through the cold plasma reactor. Compared to the Cd-0, Ni-0 and Pb-0 spectra, additional peaks are visible, which indicate the formation of various types of bonds. The peaks are summarized in Table 9. They are not unequivocally characteristic for the metals Cd(II), Ni(II) and Pb(II), which makes it difficult to identify the bonds clearly. According to [71,72], peaks 400–510 cm^−1^ in Cd-6 can be attributed to CdO. Peaks in the same region in Ni-6 spectrum are assigned to NiO and Ni-OH bonds [73,74]. No characteristic peaks related with Pb(II) are observed for Pb-6, which confirms Pb(II) was removed from the solution. Other peaks in the spectra are present in all samples of Cd-6, Ni-6 and Pb-6, which indicates the formation of bonds between elements naturally present in the tested water. Apart from the peaks related with water (~3300 cm^−1^, 1640 cm^−1^ and 620 cm^−1^), there are other peaks indicating vibrations of OH (660 cm^−1^—out-of-plane bending; 1236 cm^−1^—in-plane bending). The large peak in region 1300–1500 cm^−1^ can be decomposed into two peaks: around 1327–1361 cm^−1^ and 1397–1456 cm^−1^. The first one can be attributed to CO_3_^2−^ [73] or CH_2_ wagging [75], whereas the second can may indicate C-H bending or O-H bending [70,75]. The peaks in region 800–900 cm^−1^ are assigned to C-O-O vibrations, and those in region 1000–1150 cm^−1^—to C-O bond [70,71,75]. Small peaks in region 2800–3000 cm^−1^ are related to CH_2_ stretching [75]. This suggests that some organic substances are created during the plasma reactor operation, which agrees with the observation of the increased value of TOC (see Table 7).

The samples marked Cd-6s, Ni-6s and Pb-6s were taken from the sludge after six passes. The spectra of Cd-6s and Ni-6s are similar to those for Cd-6 and Ni-6, respectively. This is because the collected sediment was very diluted due to its small amount. Therefore, control sediments were made (NaOH was added to the prepared Cd(II) and Ni(II) solutions not subjected to plasma treatment to precipitate Cd(II) and Ni(II))—these samples are marked Cd-cs and Ni-cs. Apart from the peaks related with CdO and NiO in region 400–510 cm^−1^, there are also very sharp peaks 3600 cm^−1^ and 3639 cm^−1^, which can be attributed to Cd(OH)_2_ and Ni(OH)_2_, respectively [70,76]. Comparing the spectra of Cd-6s with Cd-cs and Ni-6s with Ni-cs, it can be seen that some of the peaks appear in both spectra, which indicates the presence of Cd(II) and Ni(II) in Ni-6s and Cd-6s samples in the form of various types of complexes.

In the case of Pb-6s, the sediment was clearly visible, and it has a white tint. The FTIR spectrum of Pb-6s contains a distinct wide peak around 1400 cm^−1^ and several narrow small peaks: 1733 cm^−1^ (attributed to C=O bond stretching [71]), 1051 cm^−1^ (attributed to C-O bond [70]), 839 cm^−1^ (attributed to C-O-O vibrations [70]) and 678 cm^−1^ (out-of-plane bending of OH [70]). This suggest that at least part of Pb(II) could be formed in lead white (2PbCO_3_⋅Pb(OH)_2_).

**Figure 10:**
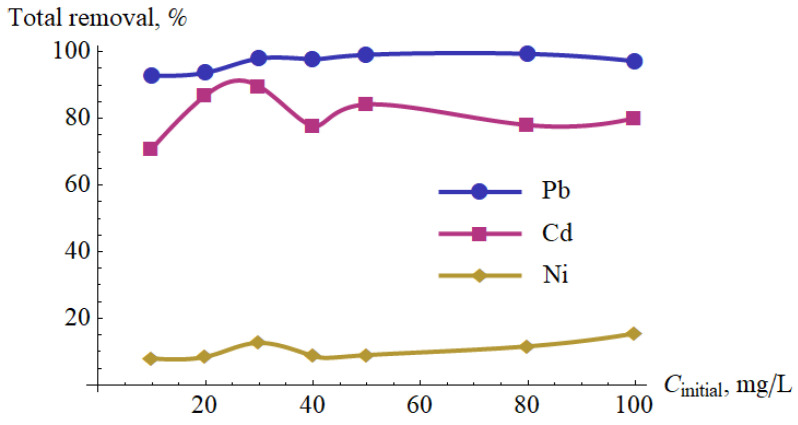
Percentage removal of Pb(II), Cd(II) and Ni(II) after one pass versus the initial concentration.

The results of the research on the effect of metal ions concentration are presented in Table 10. Based on them, the percentage removal (R) was calculated and presented in Figure 10. It follows that, in the analyzed range of concentrations, the percentage removal depended insignificantly on the initial concentration.

**Figure 11:**
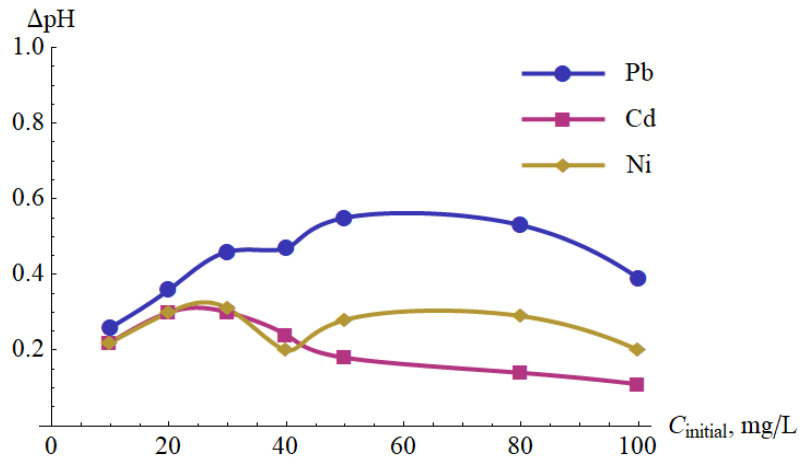
pH increase of the Pb(II), Ni(II) and Cd(II) aqueous solutions versus the initial concentration before the exposure to cold plasma and after one cycle of exposure.

Figure 11 shows the change in the pH of the tested solutions after one pass through the cold plasma reactor. In each case, an increase in pH was observed after passing through the reactor. For higher metal concentrations, there was also an overall downward trend in ΔpH (Table 10). This could be explained by the precipitation mechanism—a part of OH^−^ ions were removed from the solution in the form of metal hydroxides.

In order to estimate the energy used for water ionization, the energy consumed by the reactor was measured. In one cycle, it was on average 0.095 kWh (340 ± 30 kJ). A part of this energy was used to heat the water—the average increase in temperature was around ΔT = 2.3 ± 0.2 °C. As the volume of the solution was 5 L, the energy for heating the water in one cycle was approximately 5 kg × 4.2 kJ/(kg °C) × 2.3 °C ≈ 48 kJ. The rest of the energy—about 86%—was mainly used to ionize the water. 

**Figure 12:**
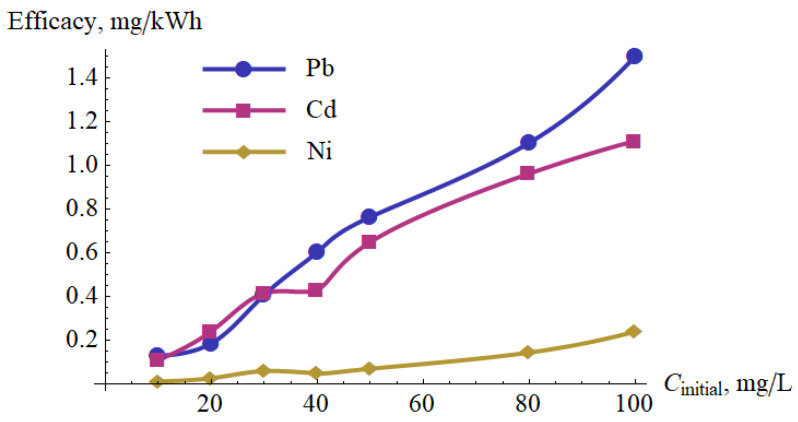
Energetical efficacy of removing metal ions.

The energetical efficacy of the metal ions removal after one pass is presented in Figure 12. In the considered range of the concentrations of metal ions, the efficacy is approximately proportional to the initial concentration.

### 3.5. Discussion on the Mechanism of Metal Ion Removal

Based on the results obtained, it follows that the increase in pH seems to be crucial for metal ions removal with NTP. The mechanism of the increase is the result of many processes occurring in the solution affected by the cold plasma. A sufficiently strong electric field causes the ionization of gas in the air bubbles in the water. Additionally, ionization is facilitated by the natural presence of a certain number of free electrons [23]. The electrons are accelerated in the electric field and gain energy large enough to ionize or excite other molecules. As a result, new electrons are released from the molecules, and UV radiation quanta are produced, which also enhance the ionization process. Examples of reactions leading to the formation of free radicals, ions and excited forms due to the interaction of an electron with water molecules are as follows [22]:

H_2_O + e^−^ → ^•^OH + H^•^ + e^−^ (5)

H_2_O + e^−^ → H_2_O^•+^ + 2e^−^ (6)

H_2_O + e^−^ → H_2_O* + e^−^ (7)

The excited water molecule H_2_O* in interaction with other water molecule can decompose into H^•^ and ^•^OH radicals or into H_2_ and O or into 2H^•^ and O. From the point of view of purifying water from metal ions, such as Pb(II), Cd(II) and Ni(II), due to the precipitation of their complexes in the form of hydroxides, the presence of OH^−^ ions in the water is important. Barrier discharges increase their content. These ions can be formed as a result of many different reactions, amongst which the most intensive are as follows:

^•^OH + e^−^ → OH^−^ (8)

H_2_O + H^•^ + e^−^ → H_2_ + OH^−^ (9)

HO_2_^−^ + e^−^ → O^−^ + OH^−^ (10)

**Figure 13:**
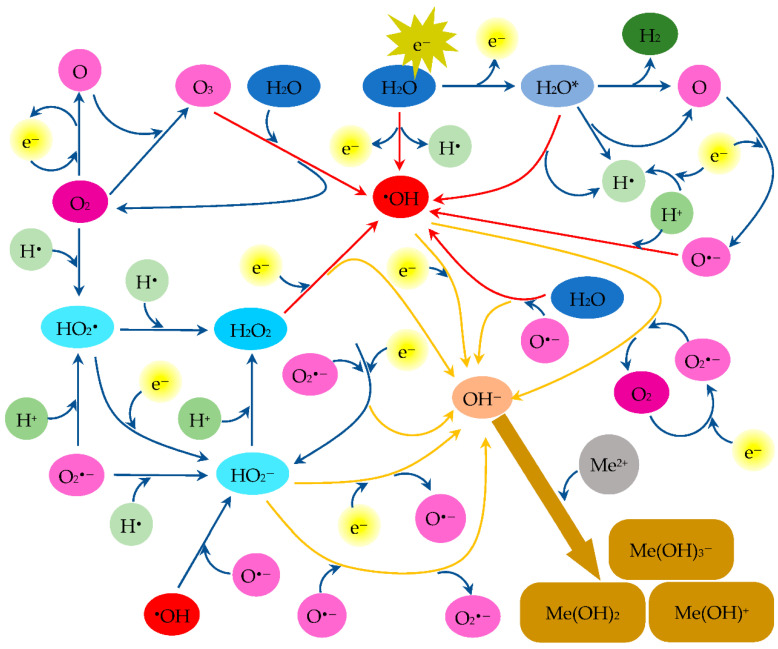
Suggested mechanism of metal ion precipitation in the aqueous solution due to nonthermal plasma (selected reactions).

Some of the reactions as well as others are illustrated in Figure 13.

The presence of oxygen significantly increases the production of OH^−^ ions. This mechanism can be described as a series of the following reactions:

O_2_ + e^−^ → O + O + e^−^ (11)

O + O_2_ → O_3_ (12)

O_3_ + H_2_O → 2 ^•^OH + O_2_ (13)

and then the reaction (8) leading to the formation of the OH^−^ ion. Some of the other oxygen-mediated reactions under the conditions under consideration are [77]:

O + e^−^ → O^•−^ (14)

O_2_ + e^−^ → O_2_^•−^ (15)

O^•−^ + H_2_O → OH^−^ + ^•^OH (16)

O_2_^•−^ + H_2_O + e^−^ → HO_2_^−^ + OH^−^ (17)

^•^OH + O_2_^•−^ → OH^−^ + O_2_ (18)

O^•−^ + H^+^ → ^•^OH (19)

O_3_ + H_2_O + 2e^−^ → 2OH^−^ + O_2_ (20)

They all increase the pH of the solution directly or indirectly. The increase in pH was observed in all considered cases (see Table 8 and Table 10, Figure 11). In such conditions, the metal ions Pb(II), Cd(II) or Ni(II) can more easily combine with OH^−^ ions, e.g.,

Pb^2+^ + OH^−^ → Pb(OH)^+^ (21)

Pb^2+^ + 2OH^−^ → Pb(OH)_2_ (22)

Pb^2+^ + 3OH^−^ → Pb(OH)_3_^−^ (23)

Similar reactions take place with the participation of Cd(II) and Ni(II) ions. It is also possible that other compounds, such as oxides or carbonates, can be formed.

### 3.6. Comparison with Chemical Precipitation

The suggested mechanism is based on pH increase, which is similar to traditional precipitation methods based on adding various agents, such as NaOH and Ca(OH)_2_. Therefore, it is worth comparing both methods (Table 11). It follows that the NTP method offers similar levels of percentage removal for particular metals as chemical precipitation with the use of low-cost precipitants. It is worth emphasizing that the NTP technology does not require the addition of other substances, often expensive (e.g., active carbons, synthesized adsorbents and precipitants, and ion exchange resins). It also does not contain expensive components subject to frequent replacement (e.g., filter membranes). It does not produce secondary waste (such as in adsorption), which is often troublesome in further processing. It is characterized by a fast and effective operation for many types of pollutants [35]. The need to supply electricity may be considered a disadvantage, but in the era of the development of photovoltaics, solutions based on solar energy are not a problem—it even can be considered as an advantage.

## 4. Conclusions

Based on the results obtained in the research, the following conclusions can be formulated:

- Cold plasma with a dielectric barrier discharge can be used to remove metal ions from aqueous solutions; in the considered case, the percentage removal was 72% for Pb(II), 88% for Cd(II) and 19% for Ni(II).
- The removal mechanism is based on an increase in pH of the solution affected by the cold plasma. Each pass of the solution through the cold plasma reactor increased pH by around 0.1–0.2; therefore, a significant effect on pH increase requires several passes (cycles) of the same solution through the cold plasma region. This may be changed by lowering the flow rate, enlarging the length of the active area or applying a cascade of several ionizers.
- The initial concentration of metal ions in the tested ranges had a small impact on the percentage removal of the metal ions—this method could be used for larger metal ions concentrations, because the energetical efficacy is largest.
- The optimization of the NTP generator settings confirmed that the most important is the flow rate, which should be as low as possible to increase the time of the impact of the cold plasma on the treated solution.
- The chemical indicators in the tested water mostly changed positively; in some cases, such as TOC, the change was undesired, but remained within the permissible limits.
- The total number of microorganisms dropped significantly after the cold plasma treatment, which is in agreement with the literature.

## Figures and Tables

**Table 1:** Values of flow rate, modulation frequency and duty cycle in the CCI method.

Variable	Unit	Normalized Variable	−*α*	−1	0	1	*α*
Flow rate	dm^3^/min	$x_{1}$	0.5	0.9	1.5	2.1	2.5
Modulation frequency	Hz	$x_{2}$	100	161	250	339	400
Duty cycle	%	$x_{3}$	10	26	50	74	90

**Table 2:** Physical and chemical indicators of the tested water along with the permissible values of pollutants in surface waters.

Indicator	Unit	Determined Values	Reference Value *
pH	-	7.7 ± 0.1 ÷ 7.8 ± 0.1	6.5–9.5
Turbidity	NTU	0.13 ± 0.03 ÷ 0.48 ± 0.11	<1.0
Electrolytic conductivity	µS/cm	587 ± 66 ÷ 603 ± 66	2500
Total hardness	mg/dm^3^ CaCO_3_	282 ± 34 ÷ 285 ± 34	60–500
Permanganate index (oxidizability)	mg/dm^3^ O_2_	0.81 ± 0.25 ÷ 1.47 ± 0.41	5
Total organic carbon (TOC)	mg/dm^3^	1.68 ± 0.2 ÷ 1.98 ± 0.2	No unacceptable changes **
Ammonium ions	mg/dm^3^	<0.1	0.5
Nitrites	mg/dm^3^	<0.01 ± 0.002	0.5
Nitrates	mg/dm^3^	30.9 ± 3.5 ÷ 34.4 ± 3.9	50
Chlorides	mg/dm^3^	68 ± 11 ÷ 74 ± 11	250
Sodium	mg/dm^3^	7 ± 1 ÷ 5 ± 1	200
Calcium	mg/dm^3^	4.92	–
Magnesium	mg/dm^3^	0.83	125 ***
Total iron	µg/dm^3^	<40	200
Manganese	µg/dm^3^	<15	50
Cadmium	µg/dm^3^	<0.6	5
Lead	µg/dm^3^	<2.2	10
Copper	µg/dm^3^	<0.26	2
Nickel	µg/dm^3^	<5.9	20
Total chromium	µg/dm^3^	<15	50

* Reference value provided in [47]. ** Need not be determined for water production lower than 10,000 m^3^ per day. *** Permissible concentration of magnesium at sulphate concentrations lower than 250 mg/dm^3^.

**Table 3:** Microbiological characteristics of the tested water.

Indicator	Unit/Sample Volume in mL	Value [Uncertainty]	Reference Value ***
Coliform bacteria	MPN */100	0 [0 ÷ 4]	0
*Escherichia coli*	MPN */100	0 [0 ÷ 4]	0
*Enterococcus*	CFU **/100	0	0
Total number of microorganisms in 22 ± 2 °C after 72 h	CFU/1	>300	100 CFU/1 mL in the water entering the water supply network 200 CFU/1 mL at the consumer’s tap

* MPN—most probable number. ** CFU—colony-forming unit. *** Reference value specified in [60].

**Table 4:** Measurement results for pH and metal ion concentration at points in the CCI measurement scheme.

No.	$x_{1}$	$x_{2}$	$x_{3}$	Final pH			Final Concentration $C\;\left[\frac{\mathrm{mg}}{{\mathrm{dm}}^{3}}\right]$		
				Pb(II)	Cd(II)	Ni(II)	Pb(II)	Cd(II)	Ni(II)
0	-	-	-	7.47	7.46	7.50	2.30	4.80	10.0
1	−1	−1	−1	7.57	7.57	7.82	1.24	2.35	9.91
2	1	−1	−1	7.50	7.55	7.83	1.12	1.63	9.95
3	−1	1	−1	7.95	7.51	7.90	0.76	3.64	9.33
4	1	1	−1	7.87	7.55	7.98	0.92	2.13	9.65
5	−1	−1	1	7.59	7.59	7.75	1.51	1.07	9.10
6	1	−1	1	7.58	7.59	7.86	1.20	1.52	8.98
7	−1	1	1	7.70	7.67	7.78	1.10	2.56	9.44
8	1	1	1	7.72	7.47	7.91	0.99	3.34	9.31
9	−α	0	0	7.64	7.61	7.82	1.56	0.90	9.46
10	α	0	0	7.56	7.61	7.71	1.11	0.85	9.77
11	0	−α	0	7.74	7.63	7.99	0.72	1.34	9.66
12	0	α	0	7.74	7.63	8.00	0.89	1.72	9.51
13	0	0	−α	7.69	7.54	7.83	0.95	2.72	9.76
14	0	0	α	7.71	7.61	7.92	0.54	2.45	9.57
15	0	0	0	7.6	7.48	7.74	0.95	3.34	9.49
16	0	0	0	7.56	7.52	7.74	0.96	3.68	9.64
17	0	0	0	7.62	7.49	7.70	1.01	3.75	9.39
18	0	0	0	7.60	7.48	7.72	1.11	3.89	9.70
19	0	0	0	7.60	7.49	7.71	1.13	3.44	9.57
20	0	0	0	7.59	7.51	7.75	0.97	3.48	9.44

**Table 5:** Fitting parameters for metal ion removal and pH change.

Term	pH Change, ΔpH			Removal [%]		
	Pb(II)	Cd(II)	Ni(II)	Pb(II)	Cd(II)	Ni(II)
1	0.1264	0.0366	0.2330	90.21	64.27	4.67
$x_{1}$	−0.0201	−0.0132	-	0.83	-	−0.46
$x_{2}$	0.0732	-	0.0239	0.74	−4.20	-
$x_{3}$	−0.0195	0.0189	-	-	-	1.71
$x_{1}^{2}$	-	0.0303	-	−1.46	8.07	-
$x_{2}^{2}$	0.0511	0.0374	0.0871	-	5.76	-
$x_{3}^{2}$	0.0369	0.0179	0.0447	0.61	2.03	-
$x_{1}x_{2}$	-	−0.0175	-	-	-	-
$x_{1}x_{3}$	-	−0.0275	-	-	−4.33	0.76
$x_{2}x_{3}$	−0.0625	-	-	-	−1.90	−1.94
adjusted *R*^2^	0.5994	0.5267	0.6952	0.3967	0.7596	0.6039

**Table 6:** Pearson correlation coefficients between generator settings and observables.

Variable	Pb(II)		Cd(II)		Ni(II)	
	ΔpH	Removal	ΔpH	Removal	ΔpH	Removal
flow rate	−0.15	0.30	−0.19	0.06	0.09	−0.15
modulation frequency	0.56	0.26	−0.10	−0.34	0.21	0.12
duty cycle	−0.15	−0.02	0.27	0.10	−0.05	0.59
ΔpH	1.00	0.51	1.00	0.77	1.00	0.01

**Table 7:** Physical, chemical and microbiological parameters of the aqueous solutions before and after the application of nonthermal plasma.

Indicator	Unit	Value			
		Untreated Water	After 1 Pass	After 3 Passes	After 6 Passes
pH	-	7.7 ± 0.1	7.72 ± 0.1	7.99 ± 0.1	8.19 ± 0.1
Turbidity	NTU	0.13 ± 0.3	0.48 ± 0.11	0.30 ± 0.07	0.23 ± 0.05
Electrolytic conductivity	µS/cm	605 ± 66	620 ± 68	618 ± 68	616 ± 68
Total hardness	mg/dm^3^ CaCO_3_	>600 ± 34	>600	>600	531 ± 61
Permanganate index	mg/dm^3^ O_2_	0.87 ± 0.25	0.91 ± 0.26	1.43 ± 0.41	0.81 ± 0.23
TOC	mg/dm^3^	1.81 ± 0.2	5.78	6.31	8.16
Ammonium ions	mg/dm^3^	<0.1	<0.1	<0.1	<0.1
Nitrites	mg/dm^3^	<0.01	<0.01	<0.01	<0.01
Nitrates	mg/dm^3^	33.6 ± 3.8	33.5 ± 3.8	30.9 ± 3.5	34.9 ± 3.9
Chlorides	mg/dm^3^	74 ± 11	72 ± 11	72 ± 11	72 ± 11
Sodium	mg/dm^3^	6.5	5.41	5.29	5.26
Calcium	mg/dm^3^	4.92	3.62	2.94	2.74
Magnesium	mg/dm^3^	0.83	0.77	0.64	0.57
Total iron	mg/dm^3^	0.19	0.097	0.067	0.05
Manganese	mg/dm^3^	0.015	0.011	0.009	0.009
Total number of microorganisms in 22 ± 2 °C after 72 h	CFU */1	>300	40 [20–80]	16 [8–34]	5 [2–12]

* CFU—colony-forming unit.

**Table 8:** The pH of the solution, metal concentration and solution temperature for the tested solutions.

Number of Passes	pH			Concentration, mg/dm^3^			Temperature, °C		
	Pb(II)	Cd(II)	Ni(II)	Pb(II)	Cd(II)	Ni(II)	Pb(II)	Cd(II)	Ni(II)
0	7.47	7.46	7.50	2.3	4.8	9.5	21.5	21.0	21.4
1	7.73	7.68	7.72	0.71	2.9	9.2	24.0	23.5	23.4
3	7.94	7.91	7.99	0.79	1.18	8.8	27.2	26.3	27.5
6	8.15	8.10	8.19	0.64	0.57	7.7	30.0	29.1	29.6

**Table 9:** Assignment of peaks in FTIR spectra (Me stands for Cd, Ni, Pb).

Me-0	Cd-6	Cd-6s	Cd-cs	Ni-6	Ni-6s	Ni-cs	Pb-6	Pb-6s	Assignment
			430						Cd-O [71]
	455			457	457	442			ν(Ni-O) [73], Cd-O [72]
			475						Cd-O [72]
		510				513			Cd-O [72], Ni-OH [74]
		596		595		571	594		out-of-plane OH bending [70]
623	620			620	623	620	620		δ(OH) [70]
	664	662	658	660	659		661		out-of-plane OH bending [70]
		685						678	out-of-plane OH bending [70]
	822	823		822	820		820		COO [70]
			835	836	836	835	837	839	CH_2_ rocking [75], COO [70]
	854			857	856		853		COO [70]
		872		875	874		873		COO [70]
			880			879			COO [70]
	1010	1002		1006	1008				C-O [70]
	1045	1048		1045	1044		1037	1051	C-O [70]
	1133	1118		1103	1111		1123	1103	C-O [70,71]
				1153		1150			C-O stretching [70,75]
	1236	1236			1285				OH in-plane bending [70]
	1333	1327	1354	1324	1327	1340	1352	1361	CO_3_^2−^ [73], CH_2_ wagging [75]
	1397	1420	1426	1404	1401	1418	1456	1397, 1443	O-H, C-H bending, γ(CH_2_), δ(OH) [70,75]
1639	1642	1633		1641	1640	1640	1636		H_2_O bending [75]
								1733	C=O stretching [71]
		~2900		~2900		~2900	~2900		CH_2_ stretching [75]
~3300	~3300	~3300	~3300	~3300	~3300	~3300	~3300		OH stretching H_2_O [70,75]
			3600						Cd(OH)_2_ [70]
						3639			Ni(OH)_2_ [70,76]

**Table 10:** Measurement results for various concentrations of Pb(II), Cd(II) and Ni(II) after one pass through the cold plasma reactor.

Initial Concentration, mg/dm^3^	Final Concentration, g/dm^3^			Initial pH			Final pH		
	Pb(II)	Cd(II)	Ni(II)	Pb(II)	Cd(II)	Ni(II)	Pb(II)	Cd(II)	Ni(II)
10	0.71	2.90	9.20	7.47	7.46	7.50	7.73	7.68	7.72
20	1.26	2.61	18.3	7.29	7.49	7.51	7.65	7.79	7.81
30	0.61	3.13	26.2	7.19	7.52	7.52	7.65	7.82	7.83
40	0.89	8.92	36.5	7.14	7.49	7.53	7.61	7.73	7.73
50	0.47	7.91	50.5	6.95	7.47	7.51	7.50	7.65	7.79
80	0.52	17.6	70.7	6.87	7.50	7.49	7.40	7.64	7.78
100	2.86	19.9	84.5	6.93	7.45	7.47	7.32	7.56	7.67

**Table 11:** Comparison of the Pb(II), Cd(II) and Ni(II) removal efficiency with cold plasma technology and chemical precipitation.

Metal	Precipitant	Metal Concentration	Conditions	Efficiency	Reference
Pb(II)	Ca(OH)_2_	>2 mg/dm^3^	pH 11–13, >2 h, 2–3 g/dm^3^	90%	[78]
	Ca(OH)_2_	100 mg/dm^3^	pH 12	99.4%	[79]
	Ca(OH)_2_	100–600 mg/dm^3^	pH 11	75.5–95.0%	[65]
	NaOH	500 mg/dm^3^	dosed up to pH 10/11.4	90/99.9%	[80]
	Ca(OH)_2_	10 mg/dm^3^	pH 9, +casein (15 mg/dm^3^)	96%	[81]
	Ca(OH)_2_	100 mg/dm^3^	pH 12.5, 25 °C, 3 g/L, +CO_2_ (1 dm^3^/min)	100%	[82]
	CaO	100 mg/dm^3^	pH 7–11	>99%	[83]
	H_2_S	0.018–2.3 mM	pH 3.0	100–92%	[84]
	Cold plasma	10 mg/dm^3^	1 pass, flow rate 0.5 1 dm^3^/min	72%	This study
Cd(II)	Ca(OH)_2_	100 mg/dm^3^	pH 12.5, 25 °C, 3 g/L, +CO_2_ (1 dm^3^/min)	100%	[82]
	Ca(OH)_2_	10 mg/dm^3^	pH 9, +casein (15 mg/dm^3^)	96%	[81]
	NaOH	~24 g/dm^3^	dosed up to pH 12	~70%	[85]
	Na_2_S	7.5 g/dm^3^	pH 12, 25 g/dm^3^	99.9%	[85]
	Cold plasma	10 mg/dm^3^	6 passes, flow rate 0.5 dm^3^/min	88%	This study
Ni(II)	NaOH	160 mg/dm^3^	dosed up to pH 12, 85 °C	84%	[86]
	NaOH	~40 mg/dm^3^	dosed up to pH 12	~62%	[85]
	Na_2_S	6 mg/dm^3^	pH 12, 25 g/dm^3^	17%	[85]
	Cold plasma	10 mg/dm^3^	6 passes, flow rate 0.5 dm^3^/min	19%	This study

## Data Availability

Not applicable.
